# 1217. Molecular Epidemiology of *Pseudomonas aeruginosa* in Latin America: Clinical Isolates From Respiratory Tract Infection

**DOI:** 10.1093/ofid/ofab466.1409

**Published:** 2021-12-04

**Authors:** Leandro Cardinal, Cicera P Marcelino, Aline Okuma, Gustavo Mizuno, Felipe Tuon, Ana C Gales, Ana C Gales, Marina Della Negra, Thales Polis, Elisa Beirao

**Affiliations:** 1 MSD in Brazil, São Paulo - SP - Brazil, New Jersey; 2 MSD Brazil, Sao Paulo, Sao Paulo, Brazil; 3 Pontifical Catholic University of Parana, Department of Medicine, Curitiba, Parana, Brazil; 4 Universidade Federal de São Paulo, Sao Paulo, Sao Paulo, Brazil; 5 MSD, São Paulo, Sao Paulo, Brazil; 6 Hospital Mandaqui, São Paulo, Sao Paulo, Brazil

## Abstract

**Background:**

Respiratory Tract Infection (RTI) caused by *P. aeruginosa* is a common infection among hospitalized patients, with increased levels of morbidity and mortality. *This pathogen *exhibits multiple resistance mechanisms to antibiotics. We analyzed the molecular epidemiology and activity of the main therapeutic options against *P. aeruginosa* isolated from RTI in Latin America (LATAM).

**Methods:**

Isolates were collected from 36 sites in 10 countries during 2017-2019. Non-duplicate samples were consecutively collected. MICs were determined by broth microdilution and interpreted by CLSI criteria. A subset of imipenem non-susceptible isolates was selected for characterization of carbapenemase encoding genes via multiplex PCR and DNA sequencing. β-lactamase genes encoding ESBLs, carbapenemases, and plasmid-mediated AmpCs were investigated.

**Results:**

A total of 2,044 *P. aeruginosa* were collected from RTI. Overall C/T [87.8% susceptible (S)] was the most active antimicrobial tested against *P. aeruginosa* isolates followed by amikacin (85.8% S) and imipenem/relebactam (IMI/REL; 82.5% S). Other antimicrobials had less than 80% susceptibility. C/T remained the most active agent including activity against imipenem and piperacillin/tazobactam non-susceptible isolates **(Figure 1).** 583 imipenem non-susceptible *P. aeruginosa* were selected for molecular analysis (**Table 1**). Thirty (5,1%) isolates were confirmed to be producers of serine-carbapenemases [GES-5 (6 isolates); KPC-2 (24 isolates)], while 83 (14.2%) were MBL producers. KPC-2 was found in Colombia (9), Chile (6), Puerto Rico (4), Guatemala (3), and Brazil (2). GES-5 was identified in Mexico (3), Argentina (2) and Brazil (1). VIM-2 was the most common MBL encoding gene identified. IMP variants were observed in Brazil (IMP-56, IMP-1), Ecuador (IMP-13), Mexico (IMP-18), Panama (IMP-18) and Puerto Rico (IMP-18). SPM-1 was only encountered in Brazil. The production of ESBLs was low in most LATAM countries, except for Guatemala (80%) (**Figure 2**).

Figure 1. Activities of selected antimicrobial agents against 2,044 P. aeruginosa isolated from respiratory tract infections in Latin America (2017 to 2019).

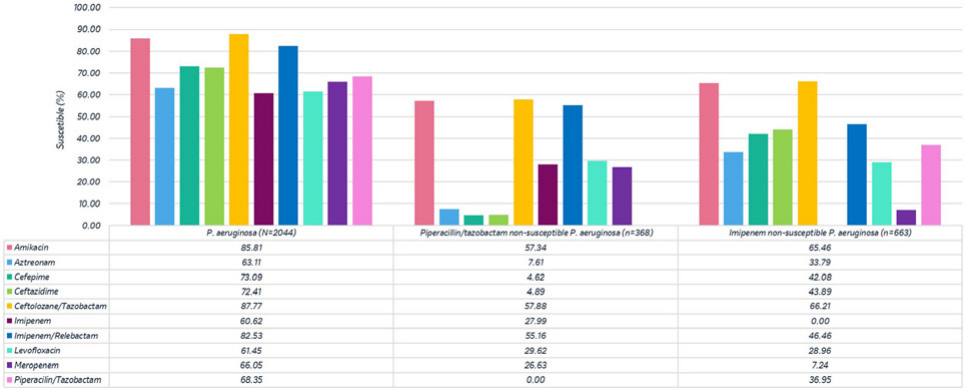

TABLE 1. Molecular analysis of imipenem non-susceptible P. aeruginosa isolates in Latin America (LATAM) from Respiratory Tract Infection (N=583).

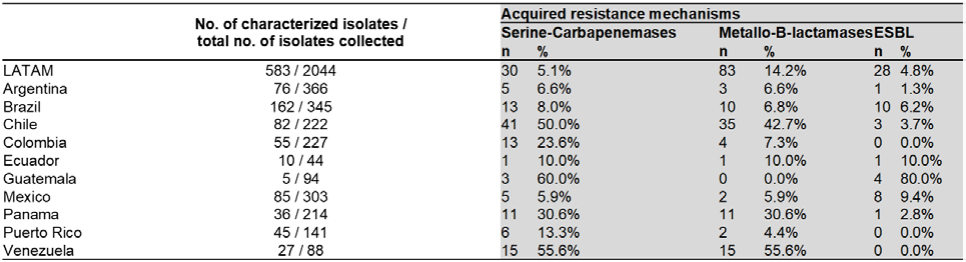

Figure 2. Carbapenemases identified in 583 imipenem non-susceptible P. aeruginosa isolated from patients with respiratory tract Infections in Latin America (LATAM).

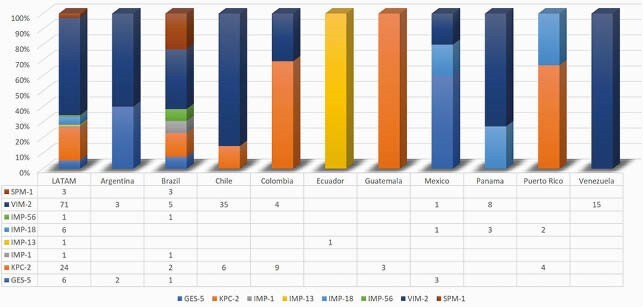

**Conclusion:**

CT, amikacin and IMI/REL showed good activity against RTI isolates and could represent effective treatment options for *P. aeruginosa* infections. The prevalence of carbapenemases-encoding genes varied geographically in LATAM.

**Disclosures:**

**Leandro Cardinal, PharmD, PhD**, **MSD** (Employee) **Cicera P. Marcelino, n/a**, **MSD** (Employee) **Aline Okuma, n/a**, **MSD** (Employee)**MSD Brazil** (Employee) **Gustavo Mizuno, PharmD**, **Merck Sharp Dohme** (Employee) **Felipe Tuon, PhD**, **Merck Sharp Dohme Brazil** (Scientific Research Study Investigator) **Ana C. Gales, MD**, **MSD** (Board Member, Advisor or Review Panel member, Speaker’s Bureau)**Pfizer** (Board Member, Consultant, Advisor or Review Panel member, Speaker’s Bureau) **Marina Della Negra, Medical Doctor**, **MSD Brazil** (Employee) **Thales Polis, Medical Doctor**, **MSD Brazil** (Employee)

